# Novel Oncology Therapeutics: Targeted Drug Delivery for Cancer

**DOI:** 10.1155/2013/918304

**Published:** 2013-10-22

**Authors:** Andreas G. Tzakos, Evangelos Briasoulis, Theresia Thalhammer, Walter Jäger, Vasso Apostolopoulos

**Affiliations:** ^1^Department of Chemistry, University of Ioannina, GR45110 Ioannina, Greece; ^2^Cancer Biobank Center, University of Ioannina, GR45110 Ioannina, Greece; ^3^Institute of Pathophysiology and Allergy Research, Center for Pathophysiology, Immunology and Infectiology, Medical University of Vienna, Vienna, Austria; ^4^Department of Clinical Pharmacy and Diagnostics, University of Vienna, Vienna, Austria; ^5^VA Consulting Services, Melbourne, VIC 3030, Australia; ^6^Victoria University, College of Health and Biomedicine, Melbourne, VIC 3021, Australia

Despite the progress in techniques for cancer prevention, detection, and treatment, as well as for increasing the public awareness in recent years, this disease is projected to become the leading cause of death worldwide. Advancements in omics, analytical procedures, and high throughput screening in the last five years have led to the realization that human diseases and especially cancer are more complex than were originally conceived. Cancer is not a static entity that can be easily monitored and manipulated. It is characterized by a dynamic and time-dependent network of constantly altered molecular and cellular interactions between players in different pathways. This network is not invariable and rigid but is constantly reshaped and altered conforming to the pliable signaling processes/responses implicated. Its complexity is apparent by the fact that the disease state is not a disruption of a single node or specific nodes in the network organism but is organism-patient dependent, thus requiring personalized perspective approaches. 

Numerous challenges hamper effective cancer treatment and development of effective drugs such as ineffective therapeutic drug concentration reaching the tumor site, life-threatening side-effects caused by nonspecific tissue distribution of anticancer agents, and acquired resistance of the cancer cell upon chemotherapy that triggers cross-resistance to a wide range of different drugs. 

Such multifactorial states require the development of very delicate approaches in the course of the drug discovery pipeline. The scientific roots of the drug development philosophy should be shifted from the traditional concept of the “magic bullet” drug (i.e., scalped for a single drug target) to the formulation of a navigated vehicle which could spatio-temporally deliver the drug in the correct location and the appropriate time. Thus, the term *targeted drug delivery* should give its place to *navigated drug delivery* since it is not only the cytotoxic drug that targets a specific cellular location but rather a vehicle that navigates the course of the loaded drug to the appropriate site of action. Such drug loaded and navigated vehicles in order to enhance the selective uptake of the cytotoxic agent by the tumor cells and spare the normal cells, should consist of a multidimensional architecture (Figures 1 and 2). The major components of these vehicles are the transporting vehicle (i.e., lipid), the cytotoxic agent that is loaded, the “programmable” navigating/targeting agent (i.e., receptor specific ligand) that enables the appropriate delivery routes to avoid toxicity on healthy proliferating cells as also ineffective concentration of the cytotoxic agent to the tumor site (Figure 2), and the “stealth” nanocarriers (biocompatibility polymers, i.e., PEG) that enhance the short plasma half-life of the drug-loaded transporting vehicle. 

In line with the challenges raised by the complexity of cancer, the aim of the present thematic issue is to provide an accumulation of innovative molecularly targeted cancer therapies and entrepreneurial methods of drug delivery in cancer (Table 1). It contains 12 papers embracing most aspects of cancer related to the exploration of active targeted nanoparticles for cancer treatment and diagnosis, including the exploitation of novel cancer drug targets, vaccine design against cancer, innovative methods for drug delivery-based on focused ultrasound or convection enhanced delivery, and mathematical modeling as an indispensable tool to analyze the transport processes and predict the outcome of anticancer treatment.

A constant developmental effort is being conducted for innovative nanoparticles to meet unmet needs in the transportation of imaging and therapeutic agents for cancer diagnosis and therapy. This happens since the nanoscale of these particles allows sculpting of diversity in their capabilities thus, enabling them to respond to a diverse array of functional requirements. Therefore, nanoparticles have been considered as appropriate vehicles to provide an ideal platform for personalized approaches to cancer diagnosis and therapy in cancer disease management. A. D. Miller presents a rigorous and descriptive overview on the status of lipid-based nanoparticles (LNPs) in cancer diagnosis and therapy. Special focus is given on LNPs that conform to the ABCD nanoparticle structural paradigm (A: active pharmaceutical ingredient, B: lipids, C: a stealth/biocompatibility polymer layer (like PEG), D: targeting layer-receptor specific ligand) and to triggered, multimodal imaging theranostic drug-nanoparticles for cancer therapy. 

Clinical attrition rates are a critical issue in drug development. In oncology, fourfold higher rates of attrition have been determined in respect of other indications. This clearly pinpoints the unmet necessity to steer for novel and traditionally unexplored drug targets to be employed in the drug discovery process towards the development of novel anticancer drugs. In this light, V. Buxhofer-Ausch et al. describe the capacity of members of the organic anion transporter family (OATP) to serve as tumor biomarkers and effective cancer drug targets. The importance of these drug targets is due to their implication on the uptake of clinically important drugs and hormones, thereby affecting drug disposition and cell penetration. An OATP-targeted therapy holds promises to combat cancer efficiently and with lesser side effects due to the tissue specific expression of different OATP members and specifically their differential expression in various cancer and normal tissues. 

Enzymes of the sulfatase pathway could offer another appealing cancer drug target. S. Lena et al. provide a comprehensive review about the expression and function of enzymes of the sulfatase pathway, particularly of steroid sulfatase (STS), in breast, endometrial, ovarian, and colorectal cancer. Furthermore, it highlighted the applicability of STS inhibitors to function as enzyme-based cancer imaging agents applied in the biomedical imaging technique positron emission tomography for the diagnosis and therapy of estrogen-sensitive cancers.

Since cancer is considered not only as a genetic but also as an epigenetic disease and tumorigenesis involves multiple genetic and epigenetic alterations that contribute to the transformation of normal cells towards a malignant phenotype, epigenetics should also be the focus of discovering novel cancer drug targets. E. Hatzimichael and T. Crook reviewed important advances in the field of cancer epigenetics and specifically provide an overview of the clinical use of epigenetics as cancer biomarkers and current progress in the utilization of epigenetic drugs in solid and blood cancers. 

Navigated delivery of cytotoxic agents to special sites or organs is a challenging issue that needs to be addressed in order to surpass systematic toxicity. These challenges become even more exigent for drug delivery in brain tumors where intrinsic difficulties are met due to the hurdles faced to cross the brain-tumor and blood-brain barriers. To establish a targeting vehicle for glioblastoma cells, T. Kasai et al. exploited the capability of chlorotoxin to selectively bind to matrix metalloproteinase-2 and other proteins on glioma cell surfaces once it was fused to human IgG-Fcs and displayed on the surface of bionanocapsules. This chlorotoxin loaded bionanocapsule showed specific affinity to the surface of glioma cells and internalized into the cytoplasmic space suggesting that is a promising drug delivery system for targeting glioblastoma.

In order to establish a target-based agent to be specifically delivered to osteosarcomas, K. Hayashi et al. focused on a lectin (Eucheuma serra agglutinin) both as a tumor-targeting agent and as an antitumor agent. Lectins are carbohydrate binding proteins that are highly specific for sugar moieties on the surface of tumor cells. The authors formulated a drug delivery system containing PEGylated Span 80 vesicles which immobilized the specific lectin. They noted that the specific system was not only selectively targeting osteosarcoma cells but was also cytotoxic to the targeted cells emphasizing the dual role adopted by lectins both as navigating and cytotoxic agents.

To achieve an effective approach for enhanced uptake of anticancer drug-loaded vehicles by tumor cells, M. W. Ndinguri et al. explored cell surface proteoglycan CD44 targeting as a way to selectively deliver therapeutic agents encapsulated inside colloidal delivery systems. CD44 has been recognized as a contributor to tumor chemoresistance and as a cancer cell and cancer stem cell biomarker due to its overexpression in cancer compared to normal cells (haematopoietic, epithelial, and neuronal cells). To target CD44, the authors used a triple-helical peptide sequence derived from type IV collagen that was incorporated in doxorubicin-loaded liposomes (composed of DSPG, DSPC, cholesterol, and DSPE-PEG-2000). The CD44-targeted liposomes were found effective in reducing tumor size, highlighting their potential to be used for selective chemotherapeutic treatment of CD44 overexpressing tumor cells.

The immune system has evolved to protect the body against microorganism invasion and thereby prevent diseases. Thus, cancer vaccines could boost the immune system to enable it to combat cancer more effectively. In vaccine development, a major aim is to induce strong, specific T cell responses. This is achieved by targeting antigens to cell surface molecules on dendritic cells (DCs) that efficiently stimulate T cell responses. The paper by V. Apostolopoulos et al. focuses on the most attractive cell surface receptors expressed on DCs to be used as targets for antigen delivery for cancer immunotherapy. The DC cell surface receptors (receptor kinases, Toll-like receptors, and C-type lectin receptors) which induce cellular responses and show promise as targets for vaccine design against cancer are highlighted. Along these lines, K.-C. Sheng et al. investigated the effect of IFN-gamma on DC functional maturation and DC meditated helper T cell activation, in the presence and absence of Toll-like receptor (TLR) ligation. The authors demonstrated an adjuvant effect of IFN-gamma on DC maturation and T cell stimulation and proposed a novel use of IFN-gamma together with Toll-like receptor agonists to enhance antigen-specific T cell responses, for applications in the development of enhanced vaccines and drug targets, against diseases, including cancer.

An alternative to the systemic drug delivery is the convection-enhanced delivery (CED) on the basis of which agents are delivered directly into the tumor and the surrounding infiltrative edges with continuous, positive-pressure infusion, thus allowing direct access to the tumor bed, achieving high local concentrations of the drug with minimal systemic absorption. The paper by J. Yun et al. focuses on the authors preclinical and clinical experience with CED in glioblastoma and highlight the challenges and potency of this methodology. Special emphasis is given onto the translational goals of this work.

M. Thanou and W. Gedroyc provided a comprehensive overview on the utilization of MRI-guided focused ultrasound (MRgFUS) as a new method of drug delivery. This methodology combines a high intensity focused ultrasound beam that can be used as an external stimulus to activate drug delivery in tissues and Magnetic Resonance Imaging system (MRI) which visualizes patient anatomy and controls the treatment by continuously monitoring the tissue effect. The advantages of being noninvasive as well as controlled and focus could establish this methodology as valuable tool in clinic to increase drug targeting and tissue specific drug delivery.

The transport of anticancer drugs and their consequence on tumor cells implicates an array of physical and biochemical processes. Since multiple steps are involved in the drug-transport, drug-release, and drug-uptake pipelines, mathematical models have become an indispensible tool to analyze the transport processes and predict the outcome of anticancer treatment. Mathematical modeling of cancer provides a tool to assist our realization on the interaction and drug-mediated perturbation of such complex processes, thus, fruitfully contributing to the optimization and refinement of drug delivery. W. Zhan and X. Y. Xu present the development of an improved mathematical model that was applied to an idealized geometry consisting of tumor and normal tissues. They predicted the efficacies of direct intravenous administration and thermosensitive liposome-mediated delivery and illustrated that thermo-sensitive liposome-mediated delivery provides a lower drug concentration in normal tissues than direct infusion of non-encapsulated drug as also a significantly higher peak intracellular concentration. These computational results furnish a projection on the effectiveness of two different treatments, within a mathematical framework, and set the basis to develop and corroborate optimized treatments with reduced risk of associated side effects as also effective tumor cell killing in a short time period of treatment.

Due to the complexity of the disease, efficient cancer therapy can emerge only upon interscience collaboration. This special issue aims to accumulate current knowledge on molecularly targeted cancer therapies and innovative methods of drug delivery in cancer. We believe that this accumulative knowledge will assist to accelerate progress developing more precise navigated drug delivery in cancer based on innovative tools.

*Andreas G. Tzakos* *Andreas G. Tzakos* *Evangelos Briasoulis* *Evangelos Briasoulis* *Theresia Thalhammer* *Theresia Thalhammer* *Walter Jäger* *Walter Jäger* *Vasso Apostolopoulos* *Vasso Apostolopoulos*

## Figures and Tables

**Figure 1:**
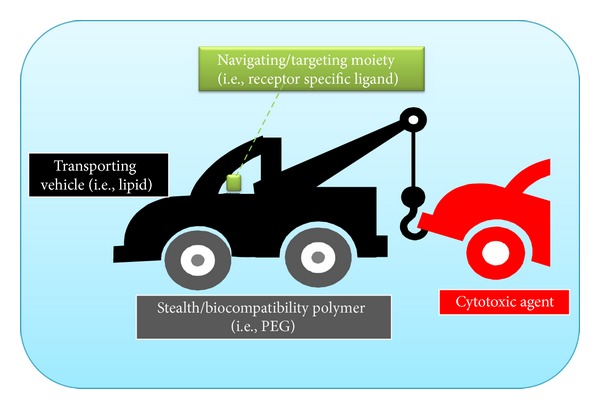
Structural architecture and mechanical analogue of a navigated drug delivery nanoparticle.

**Figure 2:**
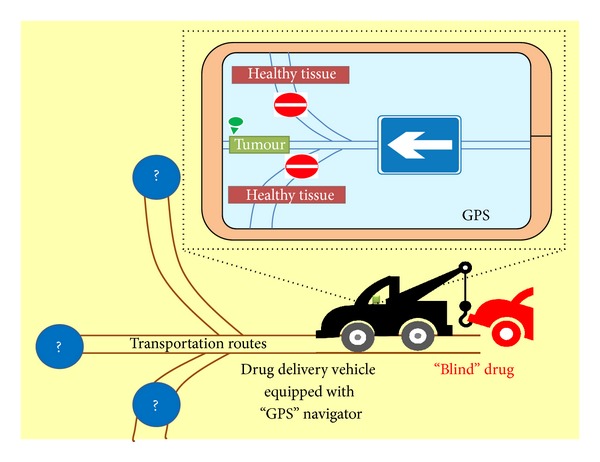
Navigated drug delivery: the drug delivery vehicle is equipped with a “programmable navigation system” that allows the transportation of the “blind” cytotoxic agent in the correct cellular location. In the absence of the drug delivery vehicle that is tagged with navigating delivery routes, toxicity is triggered on healthy proliferating cells against the anticancer agent, and ineffective therapeutic drug concentration reaches the tumor site.

**Table 1:** Some of the delivery systems appeared in this issue.

Vehicle	Vehicle description	Navigation device	Target location	Cancer type
Bionanocapsules	A bionanocapsule (BNC) is a hollow nanoparticle consisting of an approximately 100 nm diameter liposome with about 110 molecules of hepatitis B virus (HBV) surface antigen L protein embedded as a transmembrane protein.	Chlorotoxin	MMP-2 protein	Brain tumor

Enzyme-based cancer imaging agents	Dual aromatase-steroid sulfatase inhibitor (DASSI) radiotracers	^ 11^C-labelled inhibitors of steroid sulfatase (sulfamate derivatives)	Enzymes of the “sulfatase pathway”, particularly steroid sulfatase	Breast, endometrial, ovarian, and colorectal cancer

Cytotoxic drugs coupled to OATP-substrates or OATP-directed antibodies	Substrates of membrane-located OATP isoforms, selectively expressed in cancer cells, for example, microcystins as substrates for OATP1B3	Selective ligands for OATP for example, microcystins or OATP-directed antibodies in cancer cells	Organic anion transporting polypeptides (OATPs)	Gastrointestinal tract, breast, prostate, lung, brain, bladder, kidney, liver, testis

Lipid-based nanoparticles	Genuine particles (approx 100 nm in dimension) assembled from varieties of lipid and other chemical components that act collectively to overcome biological barriers (biobarriers)	*Bona fide *biological receptor specific ligands		

PEGylated Span 80 vesicles PEGylated Span 80 vesicles with immobilized ESA	Span 80 is a heterogeneous mixture of sorbitan mono-, di-, tri-, and tetra-esters	Lectin-sugar binding protein (Eucheuma serra agglutinin)	Sugar chains on the tumor cell-surface	Osteosarcoma

Doxorubicin (DOX)-loaded liposomes	Liposomes composed of DSPG, DSPC, cholesterol, and DSPE-PEG-2000	A triple-helical sequence derived from type IV collagen	CD44/chondroitin sulfate proteoglycan	CD44-overexpressing tumor cells

Mannan	Oxidized or reduced mannan, a poly-mannose, conjugated to cancer antigen	Mannan	Mannose receptor	Adenocarcinoma

